# Bioactive lipid screening during respiratory tract infections with bacterial and viral pathogens in mice

**DOI:** 10.1007/s11306-022-01898-4

**Published:** 2022-06-10

**Authors:** Daniel Schultz, Fabian Cuypers, Sebastian B. Skorka, Jan Rockstroh, Manuela Gesell Salazar, Jakob Krieger, Dirk Albrecht, Uwe Völker, Sven Hammerschmidt, Michael Lalk, Nikolai Siemens, Karen Methling

**Affiliations:** 1 Institute of Biochemistry, University of Greifswald, Greifswald, Germany; 2 Department of Molecular Genetics and Infection Biology, University of Greifswald, Greifswald, Germany; 3 Department of Functional Genomics, University Medicine Greifswald, Greifswald, Germany; 4 Zoological Institute and Museum, Cytology and Evolutionary Biology, University of Greifswald, Greifswald, Germany; 5 Institute of Microbiology, University of Greifswald, Greifswald, Germany

## Abstract

**Introduction:**

Respiratory tract infections are a worldwide health problem for humans and animals. Different cell types produce lipid mediators in response to infections, which consist of eicosanoids like hydroxyeicosatetraenoic acids (HETEs) or oxylipins like hydroxydocosahexaenoic acids (HDHAs). Both substance classes possess immunomodulatory functions. However, little is known about their role in respiratory infections.

**Objectives:**

Here, we aimed to analyze the lipid mediator imprint of different organs of C57BL/6J mice after intranasal mono-infections with *Streptococcus pneumoniae* (pneumococcus), *Staphylococcus aureus* or Influenza A virus (IAV) as wells as pneumococcal-IAV co-infection.

**Methods:**

C57BL/6J mice were infected with different pathogens and lungs, spleen, and plasma were collected. Lipid mediators were analyzed using HPLC-MS/MS. In addition, spatial-distribution of sphingosine 1-phosphate (S1P) and ceramide 1-phosphates (C1P) in tissue samples was examined using MALDI-MS-Imaging. The presence of bacterial pathogens in the lung was confirmed via immunofluorescence staining.

**Results:**

We found IAV specific changes for different HDHAs and HETEs in mouse lungs as well as enhanced levels of 20-HETE in severe *S. aureus* infection. Moreover, MALDI-MS-Imaging analysis showed an accumulation of C1P and a decrease of S1P during co-infection in lung and spleen. Long chain C1P was enriched in the red and not in the white pulp of the spleen.

**Conclusions:**

Lipid mediator analysis showed that host synthesis of bioactive lipids is in part specific for a certain pathogen, in particular for IAV infection. Furthermore, MS-Imaging displayed great potential to study infections and revealed changes of S1P and C1P in lungs and spleen of co-infected animals, which was not described before.

**Supplementary information:**

The online version contains supplementary material available at 10.1007/s11306-022-01898-4.

## Introduction

Infections of the respiratory tract are a global problem for human health (Collaborators, 2018). This is strikingly apparent in the current SARS-CoV-2 pandemic (Huang et al., 2020). Besides coronaviruses, airway infections can be caused by different bacterial and viral pathogens such as *Staphylococcus aureus*, *Streptococcus pneumoniae* (pneumococcus) (Aliberti & Kaye, 2013) and influenza A virus (IAV) (Collaborators, 2019). Moreover, bacterial and viral co-infections frequently occur, which can even aggravate the course of disease (McCullers, 2014). Host-derived bioactive lipids including eicosanoids and oxylipins are a group of lipids that have a substantial influence on the immune system (Tam, 2013; Dennis & Norris, 2015). They are derived from different ω-3 and ω-6 polyunsaturated fatty acids (PUFAs). PUFAs are released from cell membranes via hydrolysis by phospholipases. The most important PUFA precursors of oxylipin biosynthesis are arachidonic acid (AA), docosahexaenoic acid (DHA) and eicosapentaenoic acid (EPA). Major enzymes that biosynthesize oxylipins are lipoxygenases (LOX), cyclooxygenases (COX) and cytochrome P450 enzymes (CYPs). In addition, some of the oxylipins can be synthesized by transcellular biosynthesis (Fabre et al., 2002), non-enzymatic reactions or during lipid peroxidation processes (Hall & Murphy, 1998). Pro-inflammatory as well as anti-inflammatory immuno-modulatory properties are described for these substances. These include but are not limited to immune cell attraction (Nakayama et al., 2006; Bittleman & Casale, 1995), stimulation of microvascular permeability (Funk, 2001), inhibition of interleukin-6 secretion by macrophages (Kronke et al., 2009), activation of peroxisome proliferator-activated receptor (PPAR) (Wray et al., 2009), or inhibition of migratory ability of immune cells (Serhan, 2014). HPLC-MS/MS techniques are widely used for the separation and detection of these lipid mediators (Levison et al., 2013; Astarita et al., 2015).

In addition to the described bioactive lipids originated from PUFA conversion, also mediators from the sphingolipids, sphingosine 1-phosphate (S1P) and ceramide 1-phosphate (C1P), are involved in immune system related processes (Cuvillier et al., 1996; Arana et al., 2013; Simanshu et al., 2013). The bioactive lipids S1P and C1P are able to induce eicosanoid synthesis through the activation of cytosolic phospholipase A_2_ (Pettus et al., 2004) and COX-2 (Pettus et al., 2005) respectively. The sphingolipid derivative C1P has pro-inflammatory properties which include mast cell degranulation (Mitsutake et al., 2004) and activation of cell migration. The latter have been described for murine macrophages (Granado et al., 2009) and human monocytes (Arana et al., 2013). High amounts of S1P are found in blood plasma and lymph fluid, whereas the concentration in the secondary lymphatic organs is very low. This S1P gradient is important for lymphocyte trafficking. According to the lipid maps structure database, 14 derivatives of C1P with different acyl chain lengths are described (Sud et al., 2007). Little is known about the distribution of S1P in the spleen as an immune system related organ (Ramos-Perez et al., 2015; Wang et al., 2019). The distribution of C1P and its derivatives in the spleen is so far unknown. Matrix-assisted laser ionization MS imaging (MALDI-MS-Imaging) enables the measurement of the spatial distribution of metabolites or proteins within cryo-sections of tissue samples.

The aim of this study was to analyze alterations in the eicosanoid profile of different sample types in response to respiratory tract infections with *S. aureus*, *S. pneumoniae* and IAV of C57BL/6J mice. We show that in particular infections with IAV or *S. aureus* LUG2012 led to perturbations in AA and DHA-derived oxylipins. Most perturbations were measured in the lungs, but in case of *S. aureus* LUG2012 infection elevated concentrations of 20-HETE were also found in spleen and blood plasma samples. The co-infection with *S. pneumoniae* and IAV was characterized by an accumulation of different long chain C1P derivatives in lung and spleen. The splenic C1P accumulation could further be assigned to the red pulp, a phenomenon which to our knowledge was not described before.

## Materials and methods

### Ethics statement

All animal experiments were carried out in accordance with the regulations of the German Society for Laboratory Animal Science (GV-SOLAS) and the European Health Law of the Federation of Laboratory Animal Science Associations (FELASA). All experiments were approved by the Landesamt für Landwirtschaft, Lebensmittelsicherheit und Fischerei Mecklenburg-Vorpommern (LALLFV M-V, Rostock, Germany; permit no. 7221.3-1.1-032/17).

### Bacterial and viral strains

*S. pneumoniae* 19F (EF3030), a nasopharynx isolate from a child with frequent otitis media episodes (Andersson et al., 1981, 1983), was grown on blood agar plates (Oxoid) and cultivated to mid-log phase (optical density [OD]_600_, 0.35–0.40) in Todd-Hewitt broth (Carl Roth) supplemented with 0.5% (w/v) yeast extract (Carl Roth) at 37 °C and 5% CO_2_. Colonizing *S. aureus* strain SA113 and invasive USA300 strain LUG2012 (Mairpady Shambat et al., 2015) were cultured overnight at 37 °C in casein hydrolysate and yeast extract (CCY) medium. Influenza virus A/Bavaria/74/2009 (H1N1) was propagated as described by Eisfeld et al., (2014).

### Infection of C57BL/6J mice

Groups of 4–6 female C57BL/6J mice (8–12 weeks old; Janvier Labs) were intranasally colonized or infected under ketamine/xylazine anesthesia with *S. pneumoniae, S. aureus*, or H1N1. For colonization with *S. pneumoniae* 19F (19F_C), 20 µl PBS containing 1 × 10^7^ CFU were administered. For pneumonia with *S. pneumoniae* 19F (19F_P), 1 × 10^8^ CFU in 20 µl PBS were applied. For infections with SA113, 20 µl PBS containing 1 × 10^8^ CFU were administered. Staphylococcal pneumonia with LUG2012 was induced using 20 µl PBS containing 1 × 10^7^ CFU. For viral infections, 42 µl PBS containing 100,000 PFU were used. Control mice were mock-treated with an equivalent volume of PBS. For pneumococcal-IAV co-infections, a natural model of infection was used. Therefore, mice were colonized with *S. pneumoniae* 19F for seven consecutive days followed by IAV infection. Animals were observed daily for weight and clinical score monitoring. At different time points, mice were euthanized with isoflurane and blood, spleens and lungs were harvested. Blood was collected through cardiac puncture. Plasma was obtained by centrifugation (10 min, 1,000×g) and stored for further analyses at -80 °C.

### Lipid mediator extraction

Whole frozen lungs and spleens were pulverized using a CP02 automated cryoPREP® (Covaris). Briefly, the frozen tissue was transferred into a tissue tube (Covaris, extra thick) and cooled down by dipping in liquid nitrogen for 60 s. Next, the sample was pulverized with an impact level of four. This step was repeated. 50 mg of powder were immediately extracted with 500 µl ice cold methanol (Roth®) containing 0.1% 3,5-Di-*tert*-4-butylhydroxytoluene (Sigma-Aldrich) and 500 µl ice cold water. Next, 100 µl internal standard consisting of 12-HETE-d_8_ and 13-HODE-d_4_ (both 100 ng/ml in acetonitrile; Cayman chemicals) was added. Alkaline hydrolysis was performed by adding 300 µl of sodium hydroxide (10 mol/l; Sigma-Aldrich) followed by an incubation for 30 min at 60 °C. Immediately after hydrolysis, the pH was adjusted to a value of 6 using acetic acid (10 mol/l, VWR). For EDTA plasma samples, an aliquot of 100 µl was hydrolyzed and extracted using 500 µl ice cold methanol with 0.1% 3,5-Di-*tert*-4-butylhydroxytoluene, 500 µl ice cold water, and 100 µl internal standard solution. Samples were hydrolyzed with sodium hydroxide as described for the tissue material. Afterwards, solid phase extraction was done for all samples types as previously described (Schultz et al., 2019).

### LC-MS/MS measurement of lipid mediators

Extracts were dried under nitrogen flow (TurboVap^®^ from Biotage^®^) and reconstituted in 70 µl 80% acetonitrile (Th. Geyer^®^). Dynamic multiple reaction monitoring LC-MS/MS analysis was performed using an Agilent^®^ HPLC system (1200 series) coupled to an Agilent^®^ 6460 Triple quadrupole mass spectrometer with electrospray ionization source in negative mode. The separation was done with a Gemini^®^ (Phenomenex, Torrance, CA, USA) NX-C18 column (3 μm, 100 × 2 mm) and equivalent pre-column. The separation method and MS parameters were described previously (Schultz et al., 2020). Calibration curves with MS-certified standards (Cayman chemicals) for absolute quantification (range between 0.5 ng/ml and 50 ng/ml for HETEs and EETs, curve type quadratic, weighting 1/x) and deuterated internal standards were used. Eicosanoid classes without appropriate MS-certified standards (HEPE, HODE; HDHA) were normalized to the response of the internal standard and stated in arbitrary units (AU) in the plots. Agilent Mass Hunter Qualitative Analysis software and Agilent Mass Hunter Quantitative Analysis software (both version B.07.00) were used for MS data analysis.

### MALDI-FTICR-MS imaging

#### Tissue preparation

Complete mouse lungs were embedded in 1% carboxymethylcellulose (high viscosity, Sigma-Aldrich) and frozen for 24 h at -80 °C. Cryosections of embedded lungs (20 μm) and spleens (10 μm) were prepared using a Leica CM 1950 cryostat. Sections were transferred on cooled ITO coated glass slides (Bruker) and immediately lyophilized for 30 min. The MALDI matrix 9-aminoacridine (10 mg/mL from Sigma-Aldrich in 70% ethanol from Roth^®^) was sprayed on tissue sections using a HTX TM-Sprayer™ with the following parameters: N_2_ pressure 10 psi, nozzle temperature: 65 °C, solvent flow: 0.125 ml/min, drying time: 10 s, track space: 2 mm and 4 passes.

#### Measurement

For MALDI-MS imaging, a solarix XR (Bruker) mass spectrometer (FT-ICR-MS) operating in negative ionization mode was used. The analyzed mass range was between 150 and 1000 m/z and the mass of 9-aminoacridin was used for online calibration. The following ion transfer parameters were used: time of flight of 0,750 ms, 4 MHz frequency and RV amplitude of 350 Vpp. The capillary exit was − 150 V and Funnel 1 was set to 150 V. The laser focus was small with a frequency of 1000 Hz, 200 shots and a raster of 50 μm. An imaging run was done using flexImaging software (Bruker) and lipids were identified using the HMDB and lipid maps^®^ database with a mass tolerance of 5 ppm. In addition, identification of several ceramide-1 phosphate derivatives and sphingosine-1 phosphate was done with standard compounds (Cayman chemicals).

### Histology

#### Hematoxylin and eosin staining

After MALDI-MS imaging measurements, MALDI matrix was removed from the tissue sections via 60% and 80% ethanol wash for 5 and 3 min, respectively. Next, the H&E staining was performed and tissue section scans were done using a Reflecta MF5000 scanner.

#### Immunofluorescence staining and microscopy

Lung cryosections (20 μm) were fixed in ice-cold 4% formaldehyde (Roti^®^-Histofix) solution for 20 min and subsequently washed three times for 20 min with phosphate-buffered saline (PBS). Afterwards, the sections were permeabilized via 60 min incubation in PBS-TX (PBS containing 1% BSA (Sigma-Aldrich), 0.01% sodium azide and 0.3% Triton™ X-100). For incubation in the primary antibodies, PBS-TX was replaced by a solution containing 10 µg/ml rabbit anti-*S. aureus* antibody (ab20920, Abcam) or 20 µg/ml rabbit anti-*S. pneumoniae* (in house production of the S.H. laboratory) in PBS-TX for 60 min. All incubation and washing steps were directly applied in a humidity chamber. Slides were then washed five times for 2 min with PBS-TX. 30 µl of the secondary goat anti-rabbit AF488 IgG (H + L) (Thermo Fisher) combined with Phalloidin-Atto 550 (Sigma-Aldrich) and bis-Benzimide H33258 (Sigma-Aldrich) was added and samples were incubated for 60 min. Finally, the sections were washed three times with PBS-TX and two times with PBS before embedding with Mowiol 4–88 (Roth^®^).

The slides were investigated using either a fluorescence microscope (upright Nikon Eclipse 90i microscope equipped with a digital camera (Nikon DS2-MBWc) and analyzed using the NIS-Elements AR software), or a confocal laser-scanning microscope (inverted microscope: Leica TCS SP5 II). Scanning was performed sequentially with a speed of 400 Hz using an UV-diode laser with an excitation wavelength of 405 nm, an argon-laser with an excitation wavelength of 488 nm, and a DPSS-laser with an excitation wavelength of 561 nm. For detection, a 63x objective with a numerical aperture of 1.3 (HCX PL APO 63x) was used resulting in stacked images of 1,024 × 1,024 pixels with a pixel size of about 0.1 μm. The confocal microscope operated with a pinhole size of 103 μm in diameter and in steps of 0.13 μm (system-optimized to one airy unit and refractive correction for Mowiol (refraction index of 1.46). Antibody specificity was tested with uninfected control tissue samples.

### Proteome analysis

Lung tissue was homogenized with a pestle under liquid nitrogen and furthermore pulverized using a bead mill (Retsch, MM400, Haan, Germany) at 1,800 rpm for 2 min. The powder was resolved in 300 µl of UT solution (8 M urea/2 M thiourea) and sonified 3 times for 5 s on ice. Additionally, a centrifugation of the protein extract at 16,000 g at 4 °C for 1 h was performed. Protein estimation was carried out by using the Bradford assay kit (Pierce, Thermo Scientific, Bonn, Germany) and samples were stored at -80 °C. Prior to mass spectrometric analyses, 4 µg protein of each sample were reduced, alkylated, tryptic digested and desalted with µC-18 Zip Tip (Millipore Cooperation, Billerica, MA, USA) as described before (Salazar et al., 2013). Tryptic peptide solutions were analyzed by LC-MS/MS on an Ultimate 3000 nano-LC system (Thermo Fischer Scientific) coupled to a Q Exactive^™^ HFx mass spectrometer in data independent acquisition mode (Table S2). For generation of a complex spectral ion library further samples of murine lung tissue were analyzed in data dependent mode. For details see supplemental information. Data analyses and statistic tests were carried out using Spectronaut version 14.10.201222.47784 (Biognosys, Zurich, Switzerland) using a murine Uniprot database (version 2020/04) and R.

### Statistics

Lipid mediator analyses of mice organs were performed for 8–10 biological replicates and in at least 13 replicates for PBS control experiments. Data are presented as mean values ± SD. Multiple comparisons were done using Kruskal-Wallis-test with Dunn´s multiple comparison post-test. Statistics were performed using GraphPad Prism software (version 7.05). A *p*-value less than 0.05 was considered significant. MALDI-MS-imaging was performed with three replicates and discriminative m/z signals were analyzed using receiver operating characteristics (ROC) analysis tool (area under curve > 0.75) from SCiLS Lab software. Image data were normalized using total ion count normalization. For statistical proteome analyses, Benjamini-Hochberg multiple testing was applied.

## Results

### Clinical scoring and lung infiltration post different infections of mice

It was shown that low dose (1 × 10^7^ CFU) intranasal application of pneumococcal strain 19F (19F_C) results in asymptomatic colonization of mice for at least 16 consecutive days. In contrast, a high intranasal infection dose (1 × 10^8^ CFU) of the same strain induces pneumonia within two days (19F_P) (Cuypers et al., 2021). Furthermore, it was shown that infections of 19F_C mice with low pathogenic IAV are characterized by a pronounced immune response already at the asymptomatic early stage of infection. This response was equivalent to that in single pneumococcal pneumonia (Cuypers et al., 2021). Therefore, we aimed to determine the eicosanoid profile at a comparable weight loss, which was observed at 5 dpi co-infection. To track pathogen specific imprints, infections with colonizing *S. aureus* SA113 and highly invasive LUG2012 were also performed. Mice infections with SA113 were characterized by an initial increase in clinical score during the first two days post infection (dpi) and a full recovery of mice on day three (Figure S1A). In contrast, LUG2012 application resulted in pneumonia and high clinical score within 8 h post infection (Figure S1B). As previously shown, colonization of mice with pneumococcal strain 19F was characterized by an initial increase in clinical score and full recovery of the animals within five days (Figure S1C). Intranasal application of a high infection dose of pneumococci induced pneumonia accompanied by a clinical score peaking at day two post infection (Figure S1C). IAV infections of mice lead to a continuously increasing clinical score peaking at day five post infections (Figure S1D). Next, mice were colonized with *S. pneumoniae* 19F for seven days and subsequently infected with IAV for additional two days. The results confirmed previously observed phenotype of infections. Two days post viral infection, no clinical signs of pneumonia were observed (Figure S1E). Mice were sacrificed at the respective end time points and blood plasma, spleens, and lungs were harvested. Bacterial presence in the lungs was confirmed via immunofluorescence staining (Figure S2). Both, LUG2012 and 19F showed a strong invasion of the lungs in staphylococcal and pneumococcal pneumonia groups, respectively. In contrast, only traces of SA113 were detected in mice lungs (Figure S2).

### Oxylipin analysis of the lungs

**Fig. 1:**
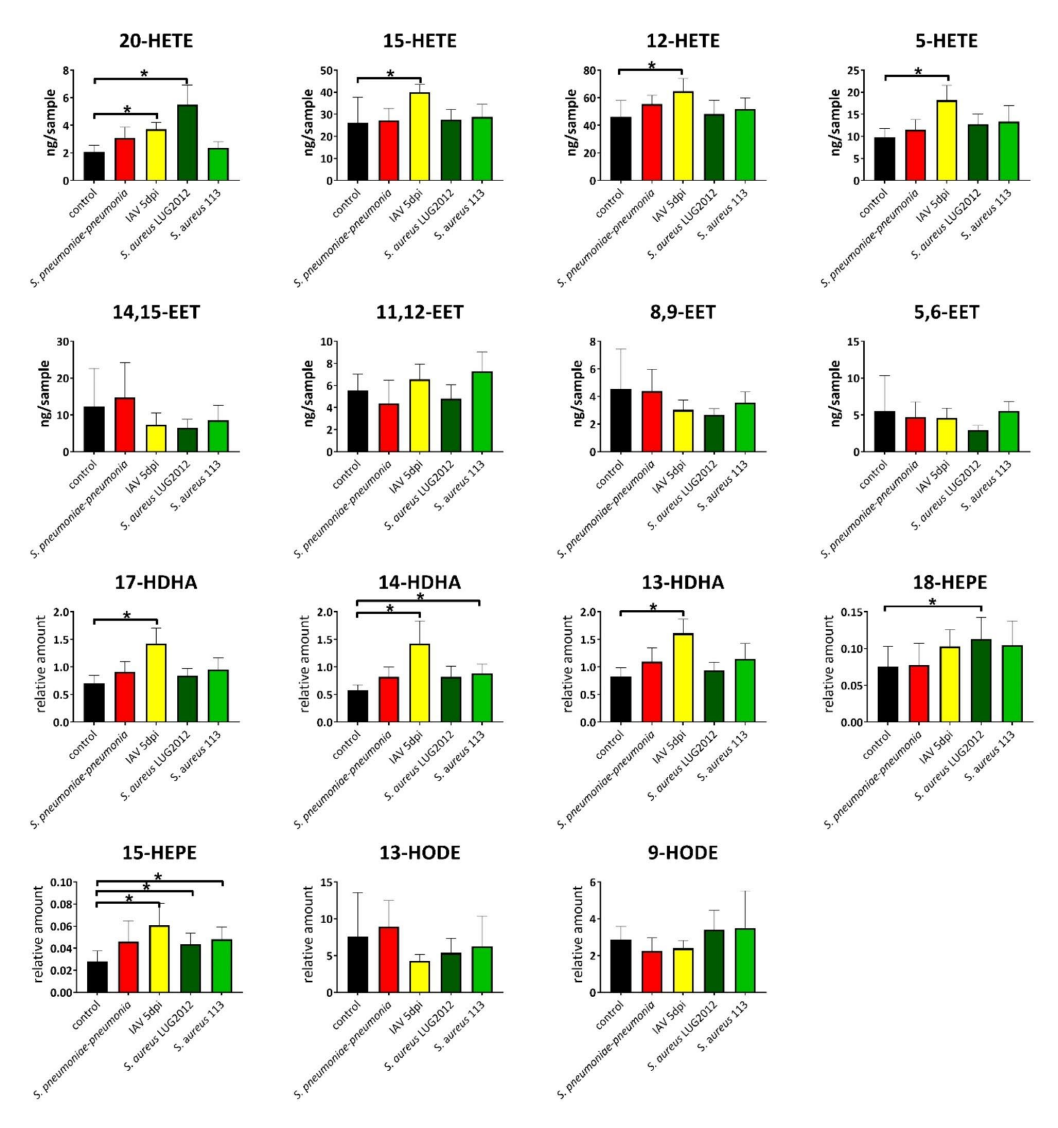
Lung oxylipin amounts in response to bacterial and viral single-infections: control (black), *S. pneumoniae* induced pneumonia (red), IAV (yellow), *S. aureus* strain LUG2012 (dark green) and strain SA113 (light green). The bars denote mean values ± standard deviations. The level of significance was determined using Kruskal-Wallis test with Dunn´s multiple comparison test (controls (n = 13) and infections (n ≥ 8)). *P*-values less than 0.05 were considered significant and are indicated by asterisks. Oxylipin amounts were normalized to the sample weight of 100 mg

First, we aimed to profile local eicosanoid composition in response to single bacterial or viral infections in mice lungs (Fig. 1). In general, major changes were observed in IAV infections. In contrast, only minor or no effects were measured in staphylococcal or pneumococcal infections, respectively (Fig. 1). Enhanced levels of mainly LOX-derived lipid mediators were detected in acute IAV infection. These include pro-inflammatory 5-and 20-HETE as well as anti-inflammatory 12-and 15-HETE (Fig. 1). Furthermore, enhanced levels of anti-inflammatory 13-, 14- and 17-HDHA were measured in mice lungs during an acute IAV infection. In contrast, LUG2012 staphylococcal pneumonia was characterized by enhanced levels of 20-HETE, 15- and 18-HEPE (Fig. 1). Acute pneumococcal infections had no influence on lipid mediator composition of the lungs, whereas enhanced amounts of only 15-HEPE were detected in infections with *S. aureus* SA113 strain.

**Fig. 2:**
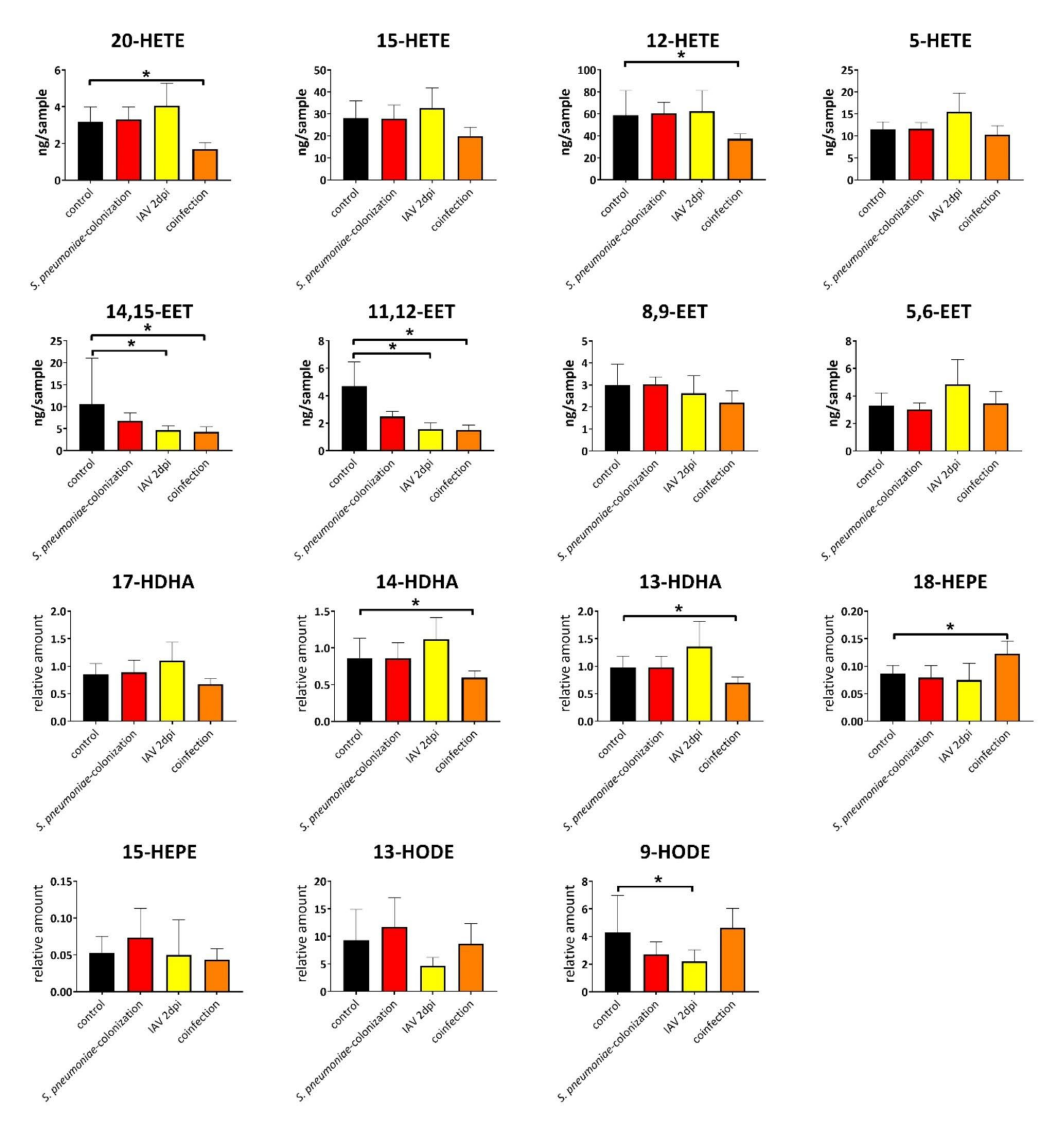
Lung oxylipin amounts in response to co-infection with *S. pneumoniae* and IAV: control (black), *S. pneumoniae* colonization (red), IAV (yellow) and co-infection (orange). The bars denote mean values ± standard deviations The level of significance was determined using Kruskal-Wallis test with Dunn´s multiple comparison test (controls (n = 13) and infections (n ≥ 6)). *P*-values less than 0.05 were considered significant and are indicated by asterisks. Oxylipin amounts were normalized to the sample weight of 100 mg

Next, we aimed to analyze eicosanoid profiles of the lungs during pneumococcal and IAV co-infections (Fig. 2). Therefore, mice were colonized with pneumococcal strain 19F for seven days and subsequently infected with IAV for two additional days. Our previous analyses revealed that even at the asymptomatic stage, co-infected mice showed a massive immune cell influx and a hyper-inflammatory response in the lungs (Cuypers et al., 2021). As shown in Fig. 2, pneumococcal colonization as well as initial single IAV infection had no significant impact on eicosanoid profile of mice lungs. In contrast, co-infections led to alterations and particularly to decreased amounts of 20- and 12-HETE. A decrease of anti-inflammatory 14,15-EET, 11,12-EET as well as 13-and 14-HDHA during co-infection was further measured. Moreover, elevated levels of 18-HEPE were exclusively detected in lungs of co-infected mice (Fig. 2).

In addition to oxylipin measurement, as part of an untargeted lung proteome analysis expression of proteins involved in oxylipin biosynthesis was investigated. According to oxylipin metabolism, increased cytosolic phospholipase A2 (cPLA_2_) levels, an enzyme responsible for the release of lipid mediator precursors from plasma membrane, were found in acute IAV infections. The analysis of lung tissue samples from IAV infection obtained from time points 4 dpi showed enhanced levels of cPLA_2_ (ratio 1.97) in comparison to the corresponding group of control animals. Moreover, enhanced amounts of the 5-LOX-activating protein (FLAP) were detected in lungs of IAV-infected animals (ratio 1.75) and infection with *S. pneumoniae* (ratio 1.94). These results are congruent with augmented amounts of the corresponding HETEs, HEPEs and HDHAs in the lungs of IAV-infected animals.

### Oxylipin analysis of the blood plasma and spleen

Next, systemic eicosanoid profiling of mice plasma and spleens was performed. Analysis of plasma samples from infected mice revealed significant perturbations in particular in response to acute bacterial mono-infections (Figure S3). Pneumococcal pneumonia was characterized by elevated levels of all four analyzed EETs and decreased amounts of 9-HODE, whereas, the *S. pneumoniae* colonization of mice had no impact on eicosanoid composition in plasma (Figure S4). Enhanced levels of 12-, 15-and 20-HETE as well as 14-and 17-HDHA were measured in mice plasma of *S. aureus* LUG2012 infected animals (Figure S3). Moreover, a decrease of both 9-and 13-HODE was measured in plasma samples of mice infected with *S. aureus* LUG2012. However, IAV infections for 2 dpi, 5 dpi and co-infection (Figure S3 and S4) were characterized by decreased amounts of 9-HODE and 13-HODE in comparison to the PBS control. Moreover, decreased EET amounts were observed for mice infected for 2 days with IAV. The analysis of the plasma samples from co-infected mice revealed reduced concentrations of 12-, 15- and 20-HETE and an enhanced amount of 18-HEPE exclusively detected in co-infections (Figure S4).

In contrast to plasma, only minor changes in lipid mediator composition of the spleens were observed. Particularly increased level of 20-HETE for *S. aureus* LUG2012 infection and enhanced amount of 12-HETE during IAV infection were noted (Figure S5). The amount of 11,12-EET was enhanced in mild SA113 infections. Furthermore, for SA113 infection decreased levels of 5-HETE and 13-HODE were observed. Co-infection led to reduced amounts of 5-HETE and 13-HDHA. Moreover, levels for all measured anti-inflammatory EETs were reduced compared to the PBS control (Figure S6). For 11,12-EET and 8,9-EET, the reduced amounts were also detected during corresponding mono-infections.

### MALDI-MS-Imaging reveals alterations of S1P and C1P under infection conditions

**Fig. 3:**
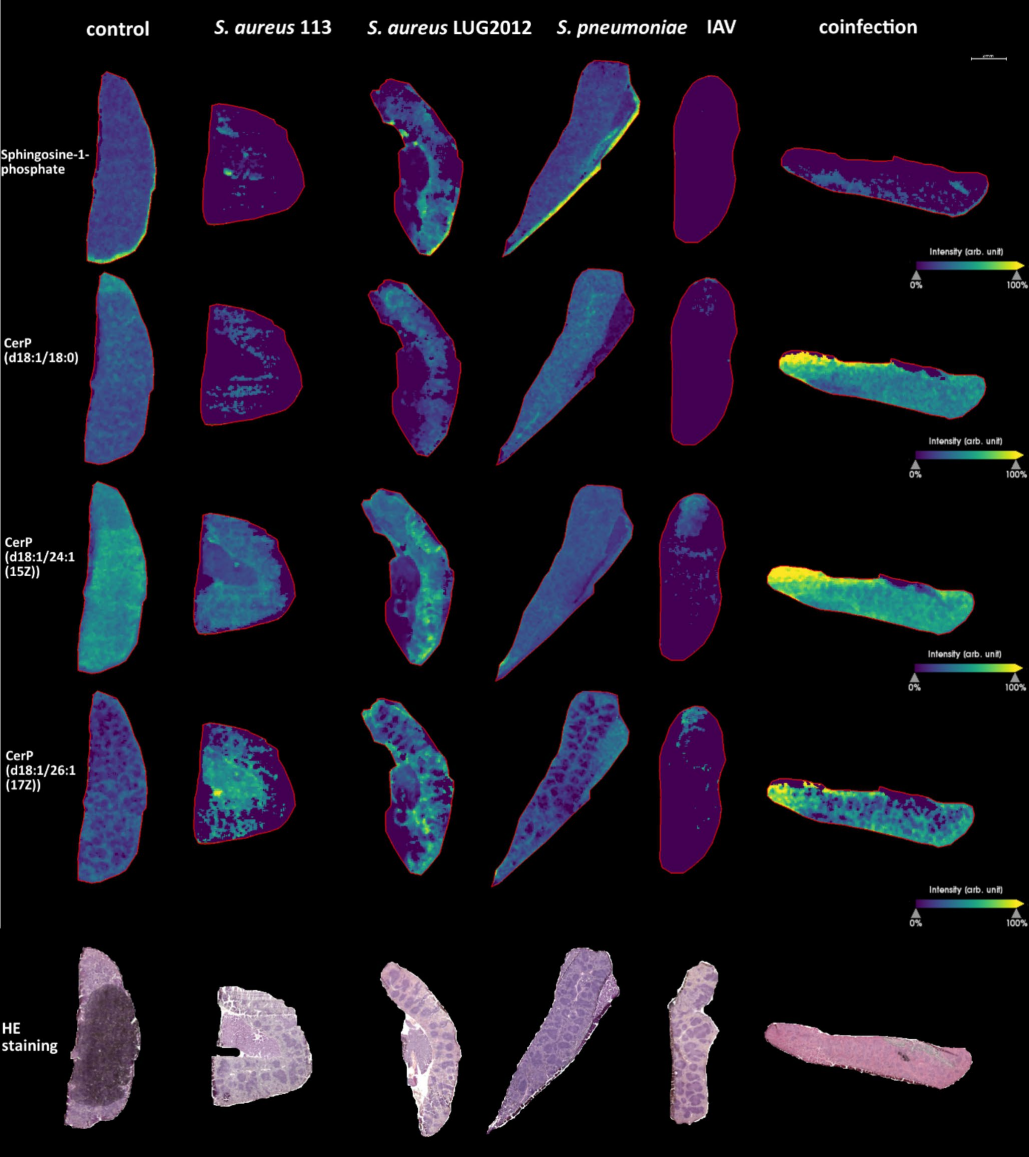
MALDI-MS-Imaging and H&E staining of spleen samples. Representative images of spleens from three measurements per condition are shown. The measurements include samples of infections with *S. aureus* strains 113 and LUG2012, *S. pneumoniae* (colonization), IAV (5 dpi) and *S. pneumoniae* – IAV co-infection. The compound-related heatmaps illustrate high intensities in yellow and low abundances in purple normalized to total ion count

In addition, we analyzed the spatial distribution of bioactive S1P and C1P (CerP) in lung and spleen samples. For statistical evaluation, ROC analysis was used and the resulting AUC values are listed in Table S1. This method is used to identify m/z values discriminating different conditions (Klein et al., 2014; Hajian-Tilaki, 2013). The measurements of the spleen samples revealed that the amount of S1P decreased during single SA113 and IAV as well as in *S. pneumoniae* - IAV co-infection (Fig. 3). Several derivatives of C1P with different acyl chain lengths exist. Three of these derivatives (C18:0, C24:1 and C26:1) were analyzed. Decreased amounts of these derivatives were detected in spleens of IAV-infected mice. In contrast, an increase was observed in co-infected animals (Fig. 3). Moreover, we observed that the amount of the long chain C1P (d18:1/26:1(17*Z*)) was higher in the red pulp as compared to the white pulp (Fig. 3). The co-infection led to increased amounts of all three C1P derivatives in the red pulp, whereas the infection with *S. aureus* LUG2012 or *S. pneumoniae* colonization had no effect.

**Fig. 4:**
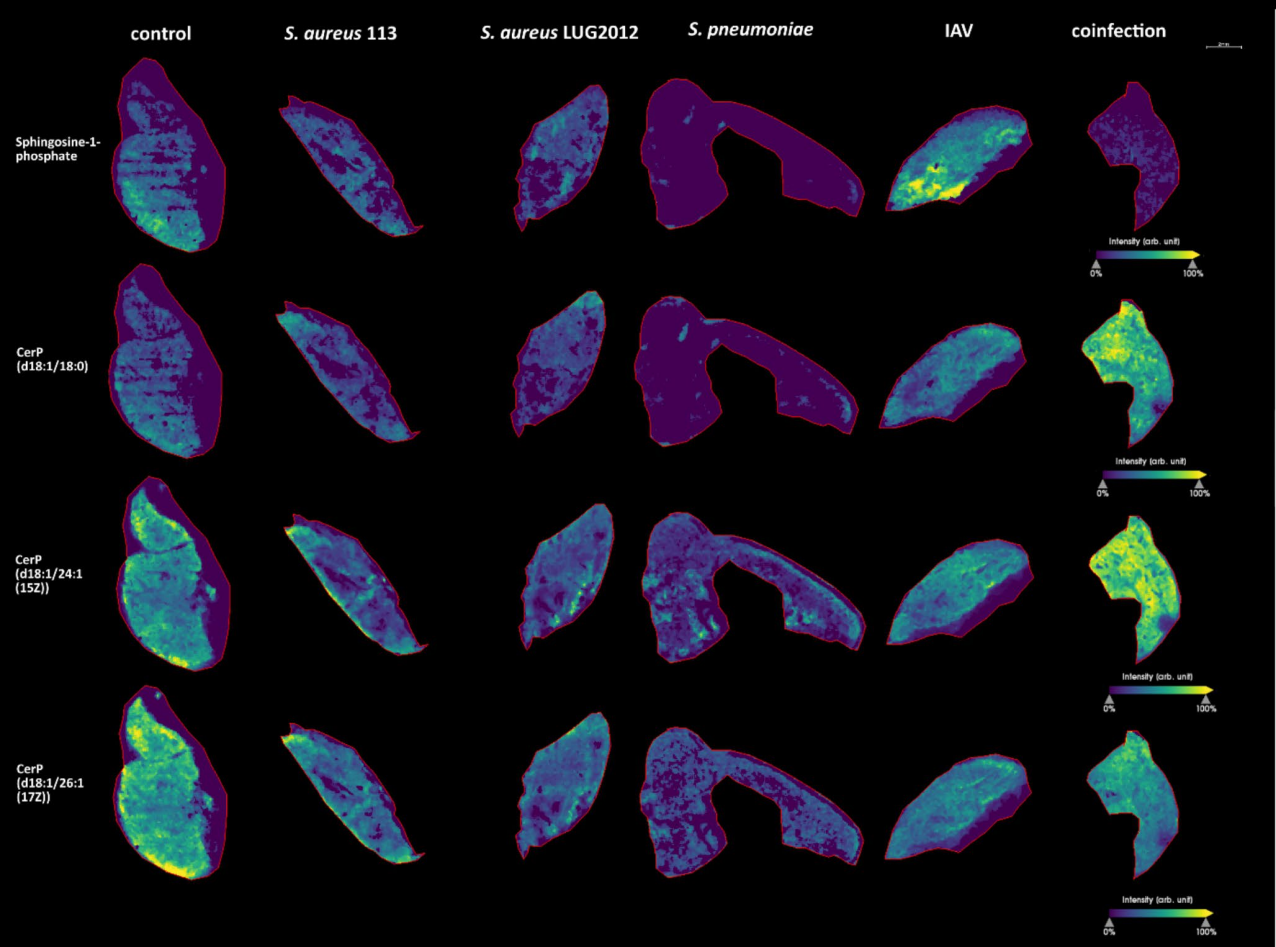
MALDI-MS-Imaging of lung samples. Representative images from three measurements per condition are shown. The measurements include samples from infection with *S. aureus* strains SA113 and LUG2012, *S. pneumoniae* (colonization), IAV (5 dpi) and *S. pneumoniae* – IAV co-infection. The compound-related heatmaps illustrate high intensities in yellow and low abundances in purple normalized to total ion count

In lungs, S1P was mostly affected in *S. pneumoniae* colonized and IAV-infected animals. Colonization with *S. pneumoniae* resulted in reduced S1P levels, whereas IAV infection led to accumulation of these substances (Fig. 4). Furthermore, a drop in C1P (d18:1/18:0) levels in *S. pneumoniae* colonized animals was noted. In contrast, enhanced amounts of C1P (d18:1/18:0) and C1P (d18:1/24:1(15*Z*)) were detected in lungs of co-infected mice (Fig. 4).

## Conclusions

The oxylipin analysis through HPLC-MS/MS combined with MALDI-MS-Imaging of different sample types from respiratory tract infected mice revealed that IAV, *S. aureus* LUG2012, and *S. pneumoniae* -IAV coinfection are responsible for the majority of changes in the bioactive lipid levels. Perturbations of eicosanoids were observed in lungs with direct pathogen contact as well as in plasma. The spleen as part of the immune system showed an increase of C1Ps with different chain lengths during co-infections restricted to the red pulp. The spleen mainly consists of two different tissue types, the capillary-rich red pulp and the white pulp functioning as part of the active immune response with B-lymphocytes and T-lymphocyte rich zones. The red pulp acts as mechanic red blood cell filtration unit, and in mice it is responsible for hematopoiesis during the entire life. On the one hand, C1P has pro-inflammatory properties including activation of cell migration, which is described for murine macrophages (Granado et al., 2009) and human monocytes (Arana et al., 2013). On the other hand, C1P seems to prevent the biosynthesis of pro-inflammatory ceramides during respiratory infection (Peng et al., 2015). To our knowledge, this is the first report of a splenic red pulp-specific distribution for long-chain C1P. The accumulation of C1Ps in the red pulp during a pneumococcal colonization followed by IAV infection was not described before and not observed for the corresponding single infections. Moreover, the impact of co-infection on C1P levels could be confirmed for two of three detected C1Ps through similar accumulations in the lung and may suggest a local and systemic host immune reaction. We demonstrated that MALDI-MS-Imaging is a useful tool to analyze sphingolipid distribution in the spleen with 9-aminoacridine as matrix and can be an alternative to laser microdissection (Wang et al., 2019). Nevertheless, further investigations are needed to understand the function of ceramide phosphates as signaling lipids in immune response to infections.

In contrast, S1P levels in lung and spleen showed a decrease under co-infection conditions. S1P is mainly known for its pro-inflammatory properties including the activation of eicosanoid synthesis through activation of COX-2 (Pettus et al., 2005). S1P is not distributed equally among organs and body fluids. High amounts of S1P are found in blood plasma and lymph, whereas the concentration in the secondary lymphatic organs is very low. This S1P gradient is important for lymphocyte trafficking (Baeyens et al., 2015). Also, S1P regulates cell migration within the spleen and shows high abundance in the marginal zone but low abundance in the white pulp as well as many regions of the red pulp (Ramos-Perez et al., 2015). The decrease during infections with IAV and corresponding co-infection may be beneficial for facilitating immune cell migration into the spleen, which seems to be independent of pneumococcal infection. Lung S1P levels showed an increase for IAV infection. Indeed, the enzyme sphingosine kinase SphK, which is responsible for S1P biosynthesis can be upregulated by IAV to promote virus propagation (Vijayan & Hahm, 2014). However, an accumulation of S1P in the lung was not detected upon co-infection, which is in congruence with the effect of pneumococcal colonization. Potentially, pneumococci masked IAV-mediated S1P signature of the lungs.

Moreover, IAV infections affected the biosynthesis of AA and DHA derived oxylipins, resulting in increased HETE and HDHA levels, which was previously detected for lungs of infected pigs (Schultz et al., 2019) or mice (Morita et al., 2013; Tam et al., 2013). Proteome data supported this observation. In particular, 17-HDHA is known for its positive effect on host antibody production against IAV (Ramon et al., 2014). Beside its effect on host B cell activation, 17-HDHA is a precursor for specialized pro-resolving mediators and moreover able to inhibit viral nucleoprotein mRNA expression in human lung epithelial cells (Andersson et al., 1983). The same effect was reported for 12-and 15-HETE (Morita et al., 2013). Furthermore, 12-and 15-HETE are anti-inflammatory and able to inhibit the interleukin-6 secretion from macrophages (Kronke et al., 2009), whereas 5-HETE is a potent chemoattractant for immune cells (Bittleman & Casale, 1995). Under co-infection conditions, the HDHAs and HETEs were not found to be elevated. However, the effect of IAV on the eicosanoid levels is clearly dependent on duration of infection, which was shown by another study (Morita et al., 2013). In our case, significant increases of the oxylipins were only observed after 5 dpi. Another reason for the different eicosanoid profile after IAV mono-infection and pneumococcal-IAV co-infection might be, that bacteria masked the effect of IAV or the asymptomatic stage of infection. In line with our observations this phenomenon is also described for children with pneumococcal-IAV co-infection regarding 17-HDHA level (Anania et al., 2020).

Our study also shows a prominent role of 20-HETE in highly invasive infections with *S. aureus* LUG2012. All analyzed samples showed elevated levels of this lipid mediator. This compound is associated with sepsis and arthritis and can influence vasoconstriction and vasodilation by release of nitric oxide (Cuez et al., 2010), induce cardiac protection in sepsis and inhibit synthesis of prostanoids like PG E_2_ (Chen et al., 2018; Tunctan et al., 2013). There is evidence that the rise of 20-HETE may be associated not only with *S. aureus* but also with severe infections in general (Volzke et al., 2020), an aspect that was also observed in other studies (Cuez et al., 2010; Tunctan et al., 2012).

Acute infections with *S. pneumoniae* 19F affected EET levels in plasma. The anti-inflammatory effect described for EETs is based on inhibition of leukocyte migration and PPAR pathway activation (Wray et al., 2009; Node et al., 1999). The EET increase seems to be dose-dependent because the EET amounts were not affected under colonization conditions.

In summary, we were able to show that infections with the selected bacterial and viral pathogens influenced the lipid mediator levels of host organs. Particularly, the lung material is a promising sample type to study eicosanoid profiles in respiratory tract infections. Moreover, MALDI-MS-Imaging showed great potential to study host reactions to different types of infection, including the bioactive lipids S1P and C1P.

## Electronic supplementary material

Below is the link to the electronic supplementary material.

Supplementary Material 1

## Data Availability

The metabolomics and metadata reported in this paper are available via MetaboLights (https://www.ebi.ac.uk/metabolights/) study identifier MTBLS3699.
