# Supplementary material for: Bioactive lipid screening during respiratory tract infections with bacterial and viral pathogens in mice
Source: Metabolomics. 2022 Jun 10;18(6):39. doi: 10.1007/s11306-022-01898-4 (PMC9185708; doi:10.1007/s11306-022-01898-4)
Supplement: Supplementary file 1 — Supplementary Material 1 [file 11306_2022_1898_MOESM1_ESM.pdf]

Supplemental Material

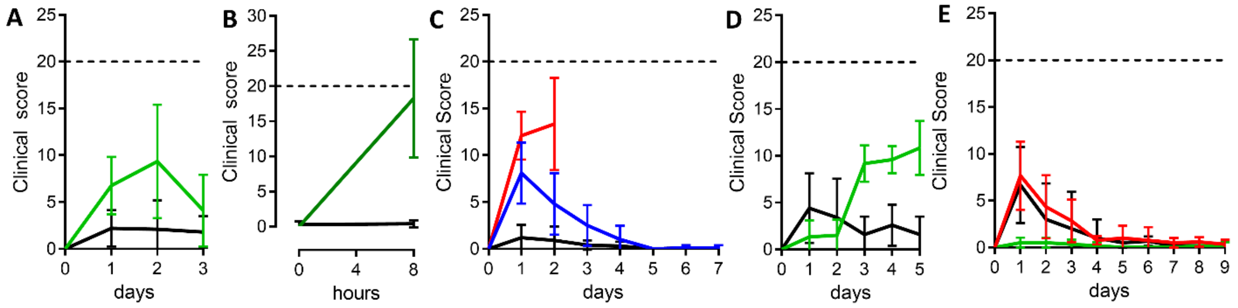

Figure S1: Clinical scores of mice intranasal infected with S. *aureus* strain 113 (1×10^8 CFU) for three days (A) and strain LUG2012 (1×10^7 CFU) for eight hours (B); *S. pneumoniae* colonization (1×10^7 CFU) for seven days (C, blue) and induced pneumonia (1×10^8 CFU) for two days (C, red), IAV for five days (D) and co-infection starting with colonization of *S. pneumoniae* for seven days (E, black) followed by IAV infection (100,000 PFU) at seven dpi for a duration of two days (green) resulting in co-infected animals (red). With exception for (E), clinical scores of control (non-infected) animals are shown in black.

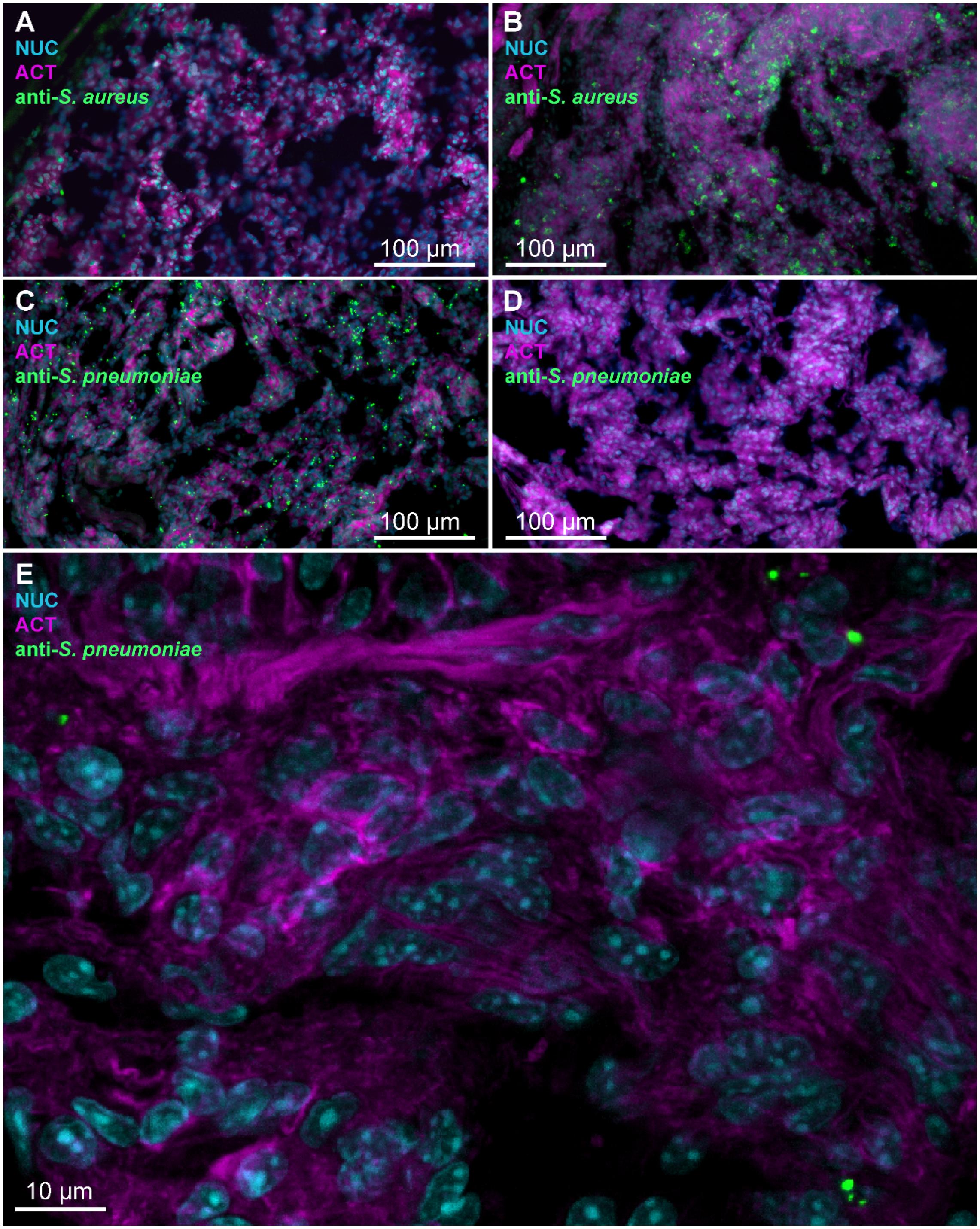

Figure S2: Representative micrographs of cryosections of lung tissue samples revealed infiltrating bacterial pathogens. The tissue samples were triple labeled with anti-*S. pneumoniae* antibody (green) or anti-*S. aureus* antibody (green) and with the nuclear counterstain HOECHST (cyan) and phalloidin staining of actin (magenta). Representative images of lung sections are displayed that were extracted from animals which were either infected with *S. aureus* strain 113 (A), LUG2012 (B), *S. pneumoniae* (C) or colonized with *S. pneumoniae* (D) and subsequently infected with IAV (E).

Table S1: Significant AUC values from ROC analysis of lung and spleen for S1P and CerPs at different infection conditions.

**bioactive lipid**	**S1P**	**CerP (d18:1/18:0)**	**CerP (d18:1/24:1(15Z))**	**CerP (d18:1/26:1(17Z))**
m/z (negative ionization)	378.241	644.502	726.580	754.612
**lung**				
control vs *S. pneumoniae*	0.800	0.788	0.778	0.834
control vs IAV	0.248	0.272	-	-
control vs coinfection	-	0.096	0.128	-
**spleen**				
control vs IAV	0.916	0.950	0.892	0.793
control vs coinfection	0.912	0.165	0.207	0.249

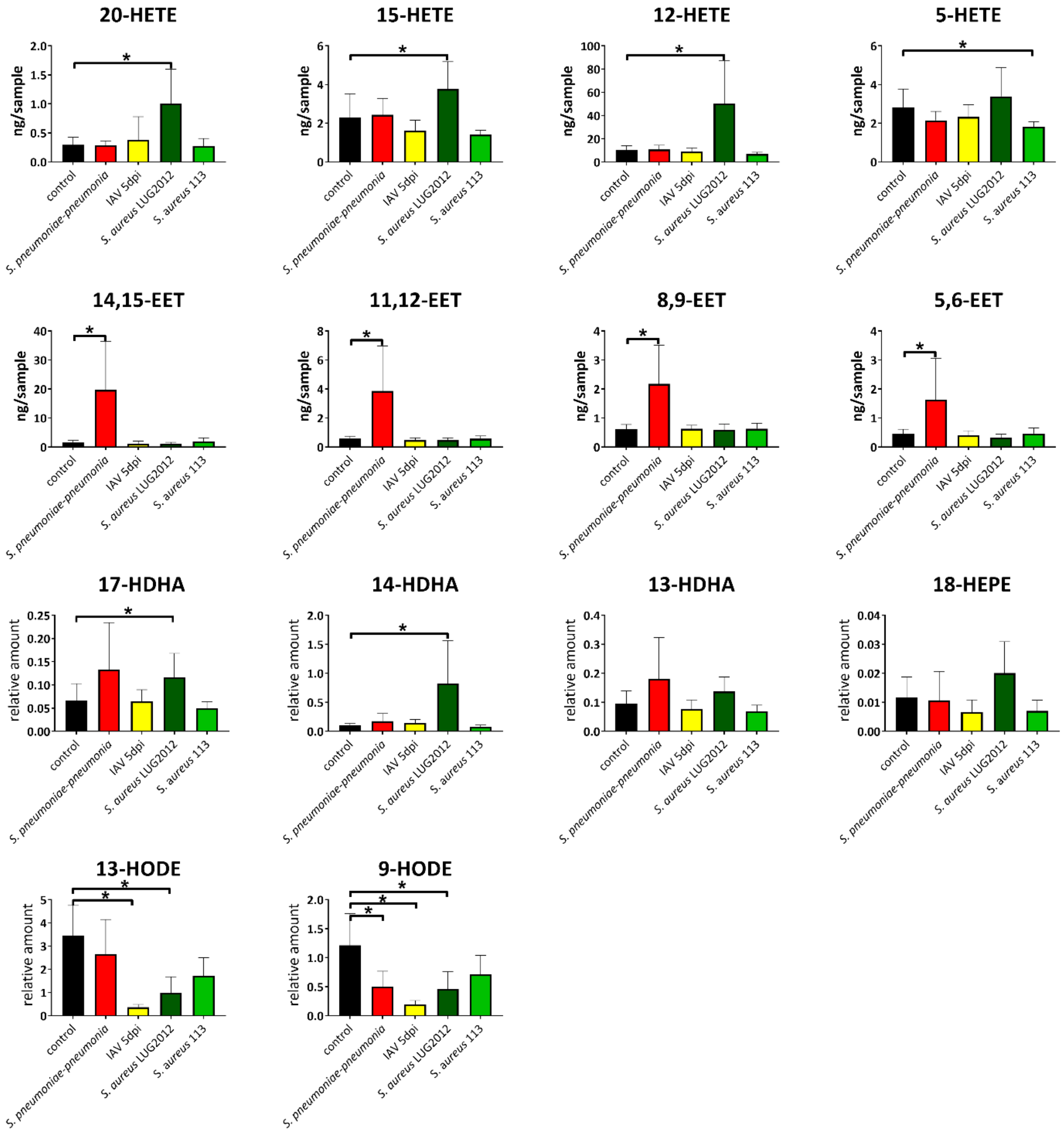

Figure S3: Plasma oxylipin amounts in response to bacterial and viral single-infections: control (black), *S. pneumoniae* induced pneumonia (red), IAV (yellow), *S. aureus* strain LUG2012 (dark green) and strain SA113 (light green). The bars denote mean values ± standard deviations. The level of significance was determined using Kruskal-Wallis test with Dunn´s multiple comparison test (controls (n =13) and infections (n≥8)). *P*-values less than 0.05 were considered significant and are indicated by asterisks. Oxylipin amounts were normalized to a plasma volume of 100 ml.

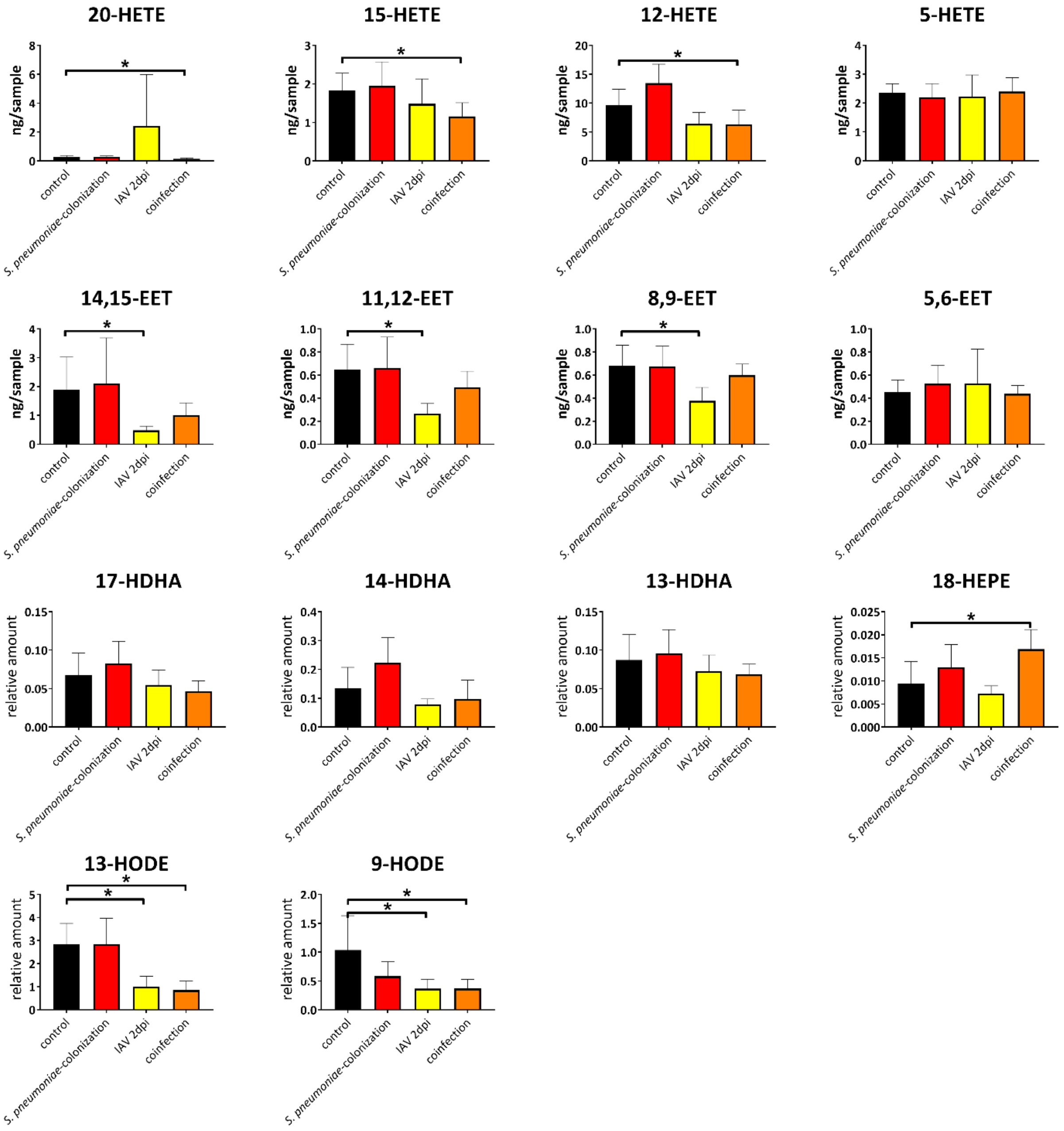

Figure S4: Plasma oxylipin amounts in response to co-infection with *S. pneumoniae* and IAV: control (black), *S. pneumoniae* colonization (red), IAV (yellow) and co-infection (orange). The bars denote mean values ± standard deviations The level of significance was determined using Kruskal-Wallis test with Dunn´s multiple comparison test (controls (n =13) and infections (n≥6)). *P*-values less than 0.05 were considered significant and are indicated by asterisks. Oxylipin amounts were normalized to a plasma volume of 100 ml.

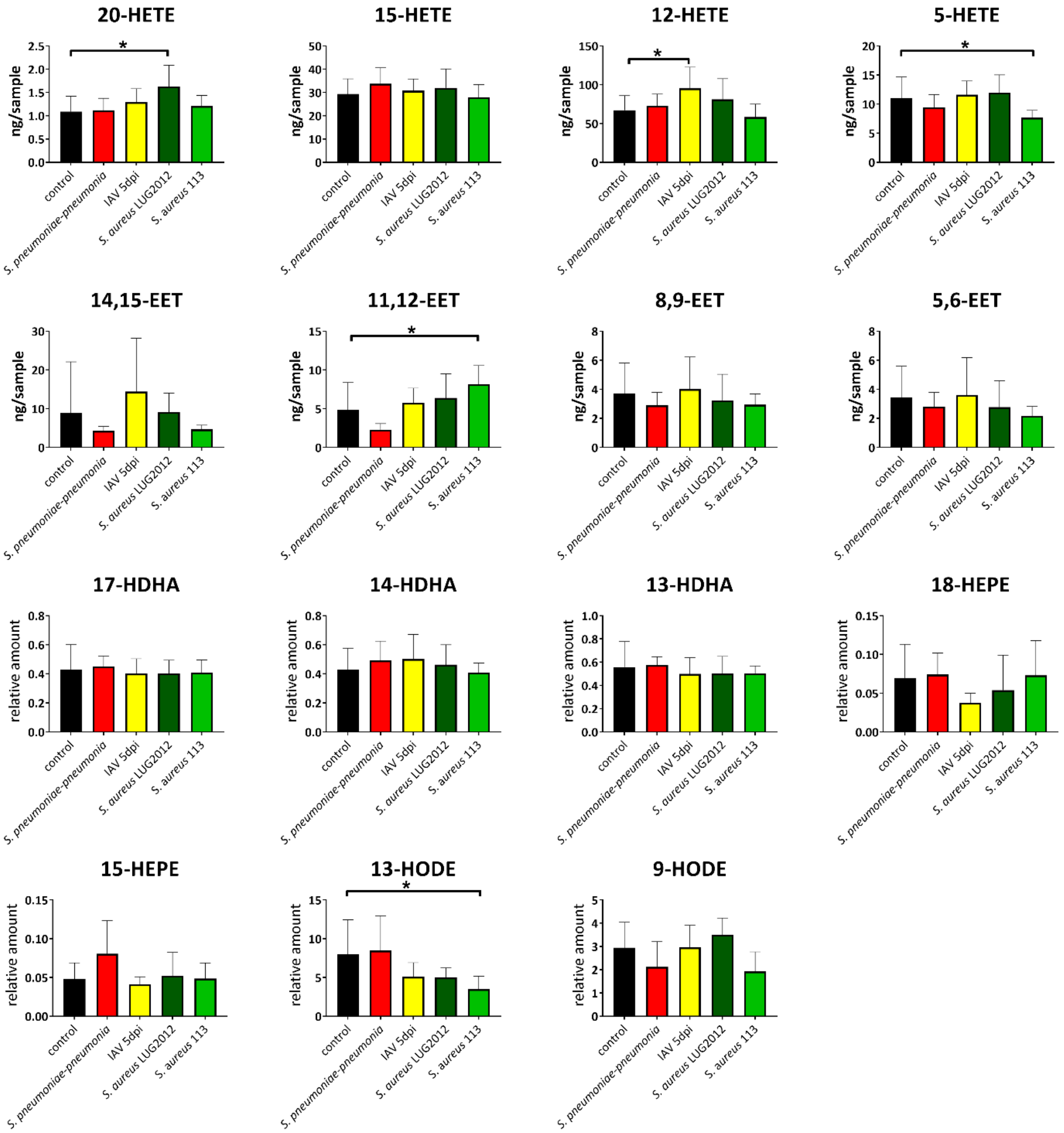

Figure S5: Spleen oxylipin amounts in response to bacterial and viral single-infections: control (black), *S. pneumoniae* induced pneumonia (red), IAV (yellow), *S. aureus* strain LUG2012 (dark green) and strain SA113 (light green). The bars denote mean values ± standard deviations. The level of significance was determined using Kruskal-Wallis test with Dunn´s multiple comparison test (controls (n =13) and infections (n≥8). *P*-values less than 0.05 were considered significant and are indicated by asterisks. Oxylipin amounts were normalized to a sample weight of 100 mg.

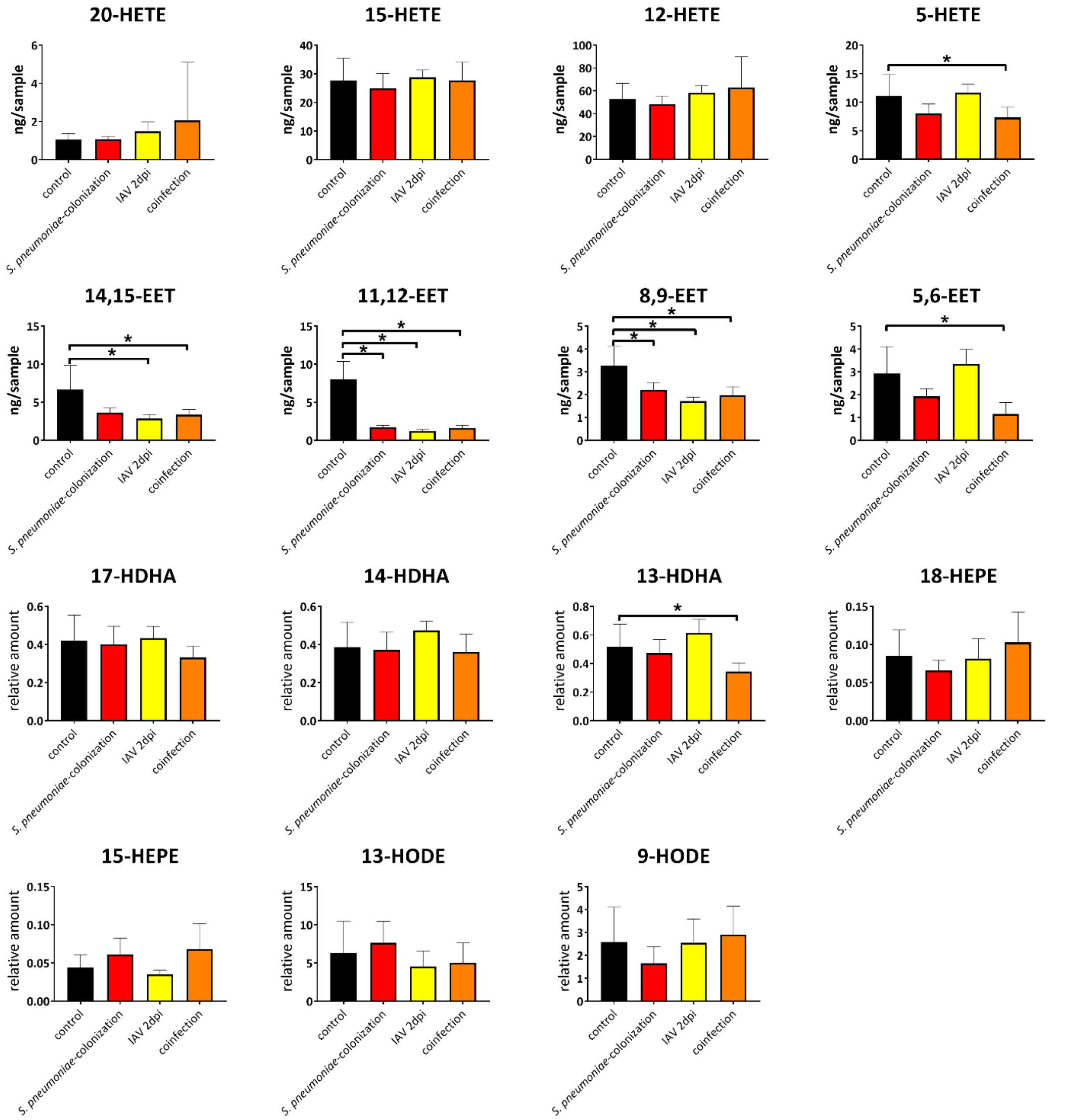

Figure S6: Spleen oxylipin amounts in response to co-infection with *S. pneumoniae* and IAV: control (black), *S. pneumoniae* colonization (red), IAV (yellow) and co-infection (orange). The bars denote mean values ± standard deviations The level of significance was determined using Kruskal-Wallis test with Dunn´s multiple comparison test (controls (n =13) and infections (n≥6). *P*-values less than 0.05 were considered significant and are indicated by asterisks. Oxylipin amounts were normalized to a sample weight of 100 mg.

Table S2: Parameters for proteome analysis.

	reversed phase liquid chromatography (RPLC)
instrument	Ultimate 3000 RSLC (Thermo Scientific)
trap column	75 µm inner diameter, packed with 3 µm C18 particles (Acclaim PepMap100, Thermo Scientific)
analytical column	Accucore 150-C18, (Thermo Fisher Scientific)<br>25 cm x 75 µm, 2,6 µm C18 particles, 150 Å pore size
buffer system	binary buffer system consisting of 0.1% acetic acid in HPLC-grade water (buffer A) and 100% ACN in 0.1% acetic acid (buffer B)
flow rate	300 nl/min
gradient	linear gradient of buffer B from 2% up to 25%
gradient duration	HFx 60min
column oven temperature	40°C
	Data dependent acquisition mode to generate a spectral library
Mass spectrometer	**Q Exactive HFx**
operation mode	data-dependent
electrospray	Nanospray Flex Ion Source
Full MS	
MS scan resolution	60,000
AGC target	3e6
maximum ion injection time for the MS scan	45 ms
Scan range	350 to 1650 m/z
Spectra data type	profile
dd-MS2	
Resolution	15,000
MS/MS AGC target	1e5
maximum ion injection time for the MS/MS scans	22 ms
Spectra data type	profile
selection for MS/MS	12 most abundant isotope patterns with charge ≥2 from the survey scan
isolation window	1,3 *m/z*
Fixed first mass	100 m/z

dissociation mode	HCD
normalized collision energy	27%
dynamic exclusion	45 s
Charge exclusion	1,>6

Data independent analyses (DIA)

Mass spectrometer	**Q Exactive HFx**
operation mode	data-independent
electrospray	Nanospray Flex Ion Source
Full MS	
MS scan resolution	120000
AGC target	3e6
maximum ion injection time for the MS scan	60 ms
Scan range	350 to 1200 m/z
Spectra data type	profile
dd-MS2	
Resolution	30,000
MS/MS AGC target	2e5
maximum ion injection time for the MS/MS scans	auto
Spectra data type	profile
selection for MS/MS	1
isolation window	56 windows m/z 13
dissociation mode	higher energy collisional dissociation (HCD)
normalized collision energy	27.5%
dissociation mode	HCD
